# Inhalable, Spray-Dried Terbinafine Microparticles for Management of Pulmonary Fungal Infections: Optimization of the Excipient Composition and Selection of an Inhalation Device

**DOI:** 10.3390/pharmaceutics14010087

**Published:** 2021-12-30

**Authors:** Khaled Almansour, Iman M. Alfagih, Alhassan H. Aodah, Fawaz Alheibshy, Raisuddin Ali, Turki Al Hagbani, Mustafa M.A. Elsayed

**Affiliations:** 1 Department of Pharmaceutics, College of Pharmacy, University of Ha’il, Hail 55473, Saudi Arabia; Kh.Almansour@uoh.edu.sa (K.A.); Fa.Alheibshy@uoh.edu.sa (F.A.); t.alhagbani@uoh.edu.sa (T.A.H.); 2 Department of Pharmaceutics, College of Pharmacy, King Saud University, Riyadh 12372, Saudi Arabia; fagih@ksu.edu.sa (I.M.A.); ramohammad@ksu.edu.sa (R.A.); 3 National Center of Biotechnology, Life Science & Environment Research Institute, King Abdulaziz City for Science and Technology (KACST), Riyadh 11442, Saudi Arabia; aaodah@kacst.edu.sa; 4 Department of Pharmaceutics, Faculty of Pharmacy, Alexandria University, Alexandria 21521, Egypt

**Keywords:** dry powder inhalation, nano spray drying, terbinafine, pulmonary aspergillosis, leucine, mannitol, Cyclohaler^®^, Handihaler^®^

## Abstract

Terbinafine is a broad-spectrum antifungal agent with therapeutic potential against pulmonary aspergillosis. The main aim of the current study was to investigate the potential of l-leucine, alone and in combination with mannitol, to improve the performance of spray-dried terbinafine microparticles for inhalation. The study also aimed to investigate the potential of the low resistance Cyclohaler^®^ and the high resistance Handihaler^®^ as inhalation devices for spray-dried microparticles. To this end, eight powder inhalation formulations of terbinafine were prepared by nano spray drying via a factorial experimental design. The formulations were evaluated in vitro for their potential to deliver the antifungal drug to the lungs using the Cyclohaler^®^ and the Handihaler^®^. Leucine was superior as an excipient to mannitol and to mixtures of leucine and mannitol. Using leucine as an excipient resulted in formulations with fine particle fractions of up to 60.84 ± 0.67% *w*/*w* and particle mass median aerodynamic diameters of down to 1.90 ± 0.20 μm, whereas using mannitol as an excipient resulted in formulations with fine particle fractions of up to 18.75 ± 3.46% *w*/*w* and particle mass median aerodynamic diameters of down to 6.79 ± 0.82 μm. When leucine was used as an excipient, using 50% *w*/*w* rather than 25% *w*/*w* ethanol in water as a spray solvent enhanced the dispersibility of the particles, with a mean absolute increase in the formulation fine particle fraction of 9.57% *w*/*w* (95% confidence interval = 6.40–12.73% *w*/*w*). This was potentially underlain by enrichment of the particle surfaces with leucine. The Cyclohaler^®^ outperformed the Handihaler^®^ as an inhalation device for the developed formulations, with a mean absolute increase in the fine particle fraction of 9.17% *w*/*w* (95% confidence interval = 8.17–10.16% *w*/*w*).

## 1. Introduction

Pulmonary fungal infections by *Aspergillus* spp. underlie a range of illnesses, which are collectively referred to as pulmonary aspergillosis [1,2]. Allergic bronchopulmonary aspergillosis and chronic pulmonary aspergillosis are two forms of pulmonary aspergillosis that principally afflict immunocompetent patients suffering from pulmonary diseases, such as chronic obstructive pulmonary disease, cystic fibrosis, or previous pulmonary tuberculosis. The fungal infection complicates the management of accompanied pulmonary diseases. Invasive pulmonary aspergillosis is a life-threatening form of pulmonary aspergillosis that mainly afflicts immunocompromised patients. Reports of pulmonary aspergillosis associated with severe acute respiratory syndrome coronavirus 2 (COVID-19) infection have recently raised concerns about the contribution of the fungal infection to the mortality of the viral disease [3]. Management of pulmonary aspergillosis typically involves systemic antifungal therapy, which is often associated with serious side effects. Administration of antifungal drugs by inhalation offers a potentially more effective and safer approach for the management of pulmonary aspergillosis [4,5].

Terbinafine is a broad-spectrum antifungal agent with therapeutic potential against pulmonary aspergillosis. We previously reported engineering of inhalable terbinafine microparticles by spray drying [6]. We investigated the influences of the excipient (lactose vs. mannitol), the spray solvent (hydroethanolic vs. aqueous), and the spray drying gas inlet temperature (90 vs. 75 °C) on the characteristics of the spray-dried microparticles. The mannitol-based microparticles were promising in terms of drug loading, solid-state stability, and disintegration characteristics, but they exhibited mediocre performance in vitro (respirable fraction ≤ 17.2 ± 0.4% *w*/*w* and mass median aerodynamic diameter ≥ 6.77 ± 0.86 when aerosolized by a Cyclohaler^®^ at an air flow rate of 100 L/min).

The main aims of the current study were to gain further insights into engineering of inhalable microparticles by spray drying and to further develop inhalable terbinafine microparticles for the management of pulmonary fungal infections. More precisely, we aimed to study the potential of l-leucine, a particle dispersibility enhancer [4,7,8,9,10,11,12,13,14], to improve the performance of spray-dried terbinafine microparticles for inhalation. Following a factorial experimental design, eight formulations were prepared to investigate the potential of leucine as an excipient alone and in combination with mannitol. The study also investigated the potential of the low resistance Cyclohaler^®^ and the high resistance Handihaler^®^ as inhalation devices for spray-dried microparticles. For this purpose, the eight formulations were evaluated in vitro for their potential to deliver the antifungal drug to the lungs using the two inhalation devices.

## 2. Materials and Methods

### 2.1. Materials

Terbinafine hydrochloride was purchased from Amino Chemicals Ltd. (Marsa, Malta). d-Mannitol (ACS reagent) was from Sigma-Aldrich (Saint Louis, MI, USA). l-Leucine was from Loba Chemie Pvt. Ltd. (Tarapur, India). Hypromellose capsules (Vcaps^®^ DPI Capsules, Size 3) were kindly provided by Capsugel France S.A.S. (Colmar, France). Ethanol 96% *v*/*v* (AnalaR^®^ NORMAPUR^®^) was from VWR International S.A.S. (Fontenay-sous-Bois, France).

### 2.2. Design of Experiments

A 4 × 2 × 2 factorial experimental design was constructed to study the influences of the excipient composition (mannitol:leucine weight ratio), the spray solvent composition (ethanol concentration), and the inhalation device (high vs. low resistance) on the characteristics of the spray-dried microparticles.

### 2.3. Preparation of Formulations

The design of the spray drying experiments is given in Table 1. Spray drying was performed using a Büchi nano spray dryer B-90 (Büchi Labortechnik AG, Flawil, Switzerland) with the tall drying chamber setup in an open configuration using dehumidified air as the drying gas. The gas inlet temperature and flow rate were set to 75 °C and 135 L/min, respectively. Spray was generated using a spray mesh with 5.5-μm-diameter pores at a spray head output of 100%. The spray drying experiments were conducted in duplicates, each designed to produce, nominally, 1.0 g of powder. The formulations were stored over silica gel in a desiccator at room temperature.

### 2.4. Characterization of Formulations

#### 2.4.1. Yield, Drug Load, and Drug Content Uniformity

After quantitative collection of each spray-dried powder formulation, the yield of the spray drying experiment was calculated using Equation (1).

(1) $$\mathrm{Yield}=\frac{M_{\mathrm{Product}}\times 100\times 100}{\left[C_{M,\mathrm{Feed}}+C_{L,\mathrm{Feed}}+C_{T,\mathrm{Feed}}\right]\times M_{\mathrm{Feed}}}\;.$$

$M_{\mathrm{Product}}$ is the mass of the collected powder (product). $M_{\mathrm{Feed}}$ is the mass of the spray solution. $C_{M,\mathrm{Feed}}$, $C_{L,\mathrm{Feed}}$, and $C_{T,\mathrm{Feed}}$ are the concentrations (% *w*/*w*) of mannitol, leucine, and terbinafine hydrochloride, respectively, in the spray solution. For all the formulations, the total solute concentration in the spray solution, i.e., $C_{M,\mathrm{Feed}}+C_{L,\mathrm{Feed}}+C_{T,\mathrm{Feed}}$, was 0.75% *w*/*w*. For determination of the drug load and content uniformity, four accurately weighed 10–15 mg samples of the formulation were dissolved in adequate volumes of a solvent mixture. The solvent mixture was a 500:499:1 *w*/*w*/*w* mixture of ethanol, deionized water, and hydrochloric acid 37% *w*/*w*. The concentrations of terbinafine hydrochloride in the solutions were then determined by UV spectrophotometry at *λ* = 240 nm [6] using a Jenway 6715 UV/Vis. spectrophotometer (Bibby Scientific Ltd., Stone, United Kingdom). The loading efficiency and the drug recovery were calculated using Equations (2) and (3).

(2) $$\mathrm{Loading}\;\mathrm{efficiency}=\frac{C_{T,\mathrm{Product}}}{C_{T,\mathrm{Product},\;N}}\times 100.$$

(3) $$\mathrm{Drug}\;\mathrm{recovery}=\frac{\mathrm{Loading}\;\mathrm{efficiency}\times \mathrm{Yield}}{100}.$$

$C_{T,\mathrm{Product}}$ and $C_{T,\mathrm{Product},\;N}$ are the real and the nominal concentrations of the drug in the powder formulation, respectively.

#### 2.4.2. Particle Morphology

Particle morphology was studied by scanning electron microscopy. The test powder samples were coated with platinum at 10 mA for 30 sec using a JEOL JEC-3000FC auto-fine coater (JEOL Ltd., Tokyo, Japan). The samples were then examined by a JEOL JSM-IT500HR scanning electron microscope (JEOL Ltd., Tokyo, Japan) at 5.0 kV. Images were captured at a 3.0 kX magnification.

#### 2.4.3. Crystallinity

Powder crystallinity was studied by differential scanning calorimetry using a Shimadzu DSC-60 Plus differential scanning calorimeter equipped with a TA-60WS thermal analysis system and the TA-60WS version 2.21 software (Shimadzu Corporation, Kyoto, Japan). The test powder samples were heated in aluminum pans under nitrogen purge from 30 °C to 330 °C at a heating rate of 3 °C/min. The measurements were conducted in duplicate.

### 2.5. Evaluation of Formulations

#### 2.5.1. Particle Disintegration and Dissolution

Particle disintegration and dissolution were studied by spectrophotometry and dynamic light scattering using a modification of the method proposed by Almansour et al. [6]. The disintegration medium was a 25 mM pH 6.6 phosphate buffer containing 0.008% *w*/*w* polysorbate 80. The composition simulates the pH of the endobronchial fluid [15] and the solubilizing effect of lung surfactants [16]. In each experiment, a 20 mg sample of the test powder formulation was dispersed in 30 mL of the disintegration medium at 37 °C. The dispersion was vortexed for 10 s and then maintained stagnant at 37 °C. At 2 and 120 min, samples were taken to estimate the amount of the drug dissolved and to determine the size of undissolved drug particles. To estimate the amount of the drug dissolved, the optical density of the dispersion at *λ* = 200–400 nm was recorded using a Jenway 6715 UV/Vis. spectrophotometer (Bibby Scientific Ltd., Stone, United Kingdom). The concentration of terbinafine hydrochloride dissolved in the dispersion was estimated from the optical density at *λ* = 284 nm after eliminating the contribution of light scattering by undissolved particles to the optical density. The contribution of light scattering was estimated by extrapolating the optical density data at *λ* = 330–400 nm, where light absorbance by terbinafine molecules is negligible, to *λ* = 284 using the power function: $S\left(\lambda \right)\approx B\lambda^{w}$ [17]. This procedure provides a satisfactory, semi-quantitative estimate of the amount of the drug dissolved and allows comparison between formulations. The size of undissolved particles was determined by dynamic light scattering at 37 °C using a Malvern Zetasizer nano ZSP operated with the Malvern Zetasizer software version 7.13 (Malvern Instruments Ltd., United Kingdom). Particle disintegration and dissolution experiments were conducted in triplicate.

#### 2.5.2. In Vitro Aerodynamic Performance (Cascade Impaction)

The formulations were evaluated in vitro using a Next Generation Impactor (Copley Scientific, Nottingham, United Kingdom), following the procedure of the United States Pharmacopeia [18]. The formulations were evaluated using two inhalation devices: a Cyclohaler^®^ (Pharmachemie B.V., Haarlem, The Netherlands; flow rate = 100 L/min, Δ*P* = 2.97 kPa, actuation time = 2.4 s) and a Handihaler^®^ (Boehringher Ingelheim, Ingelheim, Germany; flow rate = 39 L/min, Δ*P* = 4.00 kPa, actuation time = 6.15 s). A 500:499:1 *w*/*w*/*w* mixture of ethanol 96% *v*/*v*, deionized water, and hydrochloric acid 37% *w*/*w* was used as the collection solvent. For each experiment, the preseparator was filled with 20 mL of the collection solvent, and the impactor stages were not coated. In each experiment, two capsules, each filled with 20 ± 1 mg of the test formulation, were actuated. Particles deposited on the capsule shells, the inhalation device, the induction port, the mouthpiece adapter, the preseparator, and the impactor stages were collected by rinsing with the collection solvent. The concentrations of terbinafine hydrochloride in the collected samples were determined by UV spectrophotometry at *λ* = 240 nm [6] using a Biochrom Libra S22 UV/Vis. spectrophotometer (Biochrom Ltd., Cambridge, United Kingdom). Cascade impaction experiments were conducted in triplicate under controlled temperature (20 ± 2 °C) and relative humidity (40 ± 5% relative humidity).

The in vitro aerodynamic performance is expressed in terms of the emitted fraction (EF), the fine particle fraction (FPF), and the mass median aerodynamic diameter (MMAD). The emitted fraction was calculated as the ratio (% *w*/*w*) of the amount of the drug emitted from the device to the total amount of the drug collected. The fine particle fraction was calculated as the ratio (% *w*/*w*) of the amount of the drug in particles with an aerodynamic diameter smaller than 5.00 μm to the amount of the drug emitted from the device. The fine particle dose, FPD_N_, was calculated using an expression that normalizes capsule filling and dose recovery differences (Equation (4)).

(4) $${\mathrm{FPD}}_{N}=\frac{\mathrm{FPF}}{100}\times \frac{\mathrm{EF}}{100}\times \frac{\mathrm{LE}}{100}\times C_{T,N}\times D.$$

LE is the mean loading efficiency (% *w*/*w*), $C_{T,N}$ = 150 μg/mg the nominal concentration of the drug in the formulation, and *D* = 20 mg the nominal powder dose. The mass median aerodynamic diameter was calculated as the 50th percentile of the cumulative (undersize, by mass) aerodynamic size distribution of the particles collected from the impactor stages.

### 2.6. Data Analysis

OriginPro 2021 (OriginLab Corporation, Northampton, MA, USA) was used for mathematical and statistical data analysis. Two-way or three-way analysis of variance (ANOVA) and Tukey’s post hoc test were used for statistical comparison. The significance level was set to 0.05 unless otherwise stated.

## 3. Results

### 3.1. Characterization of Formulations

#### 3.1.1. Yield, Drug Load, and Drug Content Uniformity

The yields and the loading efficiencies of the spray drying experiments are presented in Table 2. The yields of the spray drying experiments exhibited high variability, with relative standard deviations of up to 25.57%. Drug contents were, however, satisfactorily uniform, with relative standard deviations of 1.39–6.50%. The excipient composition and the spray solvent composition did not significantly influence the yield (two-way analysis of variance: *p* > 0.05) but influenced the loading efficiency (two-way analysis of variance: *p* < 0.001) of the spray drying experiment. The loading efficiency decreased as the concentration of leucine in the formulation increased, whereas it increased as the concentration of ethanol in the spray solvent increased. Replacing mannitol with leucine as a sole excipient was associated with a mean absolute decrease in the loading efficiency of 23.9% *w*/*w* (Tukey’s post hoc test: 95% CI = 19.3–28.4% *w*/*w*). On the other hand, increasing the concentration of ethanol in the spray solvent from 25 to 50% *w*/*w* was associated with a mean absolute increase in the loading efficiency of 7.9% *w*/*w* (Tukey’s post hoc test: 95% CI = 5.5–10.3% *w*/*w*).

#### 3.1.2. Particle Morphology

**Figure 1:**
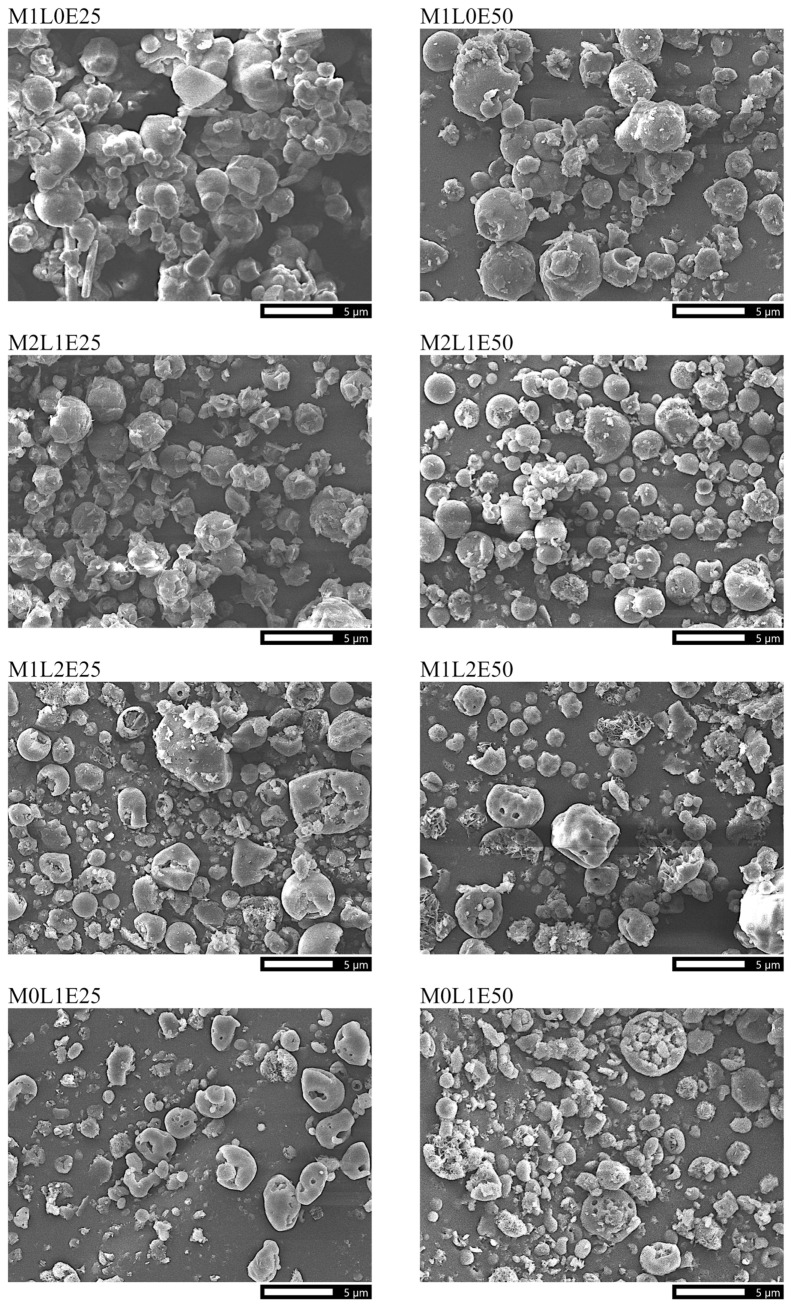
Scanning electron micrographs of the powder formulations. All micrographs were taken at the same magnification of 3 kX. The scale bar at the bottom of each micrograph represents a length of 5 μm.

Scanning electron micrographs of the formulations are presented in Figure 1. The formulations exhibited average particle diameters smaller than 5 μm. As the concentration of leucine in the formulation increased from 0.0 to 100.0% *w*/*w* of the total excipient, the formulation particles changed from relatively spherical to wrinkled, irregularly dimpled in shape, became more fragmented and surface perforated, and appeared to generally decrease in size. The spray solvent composition did not remarkably affect the morphology of the particles.

#### 3.1.3. Crystallinity

**Figure 2:**
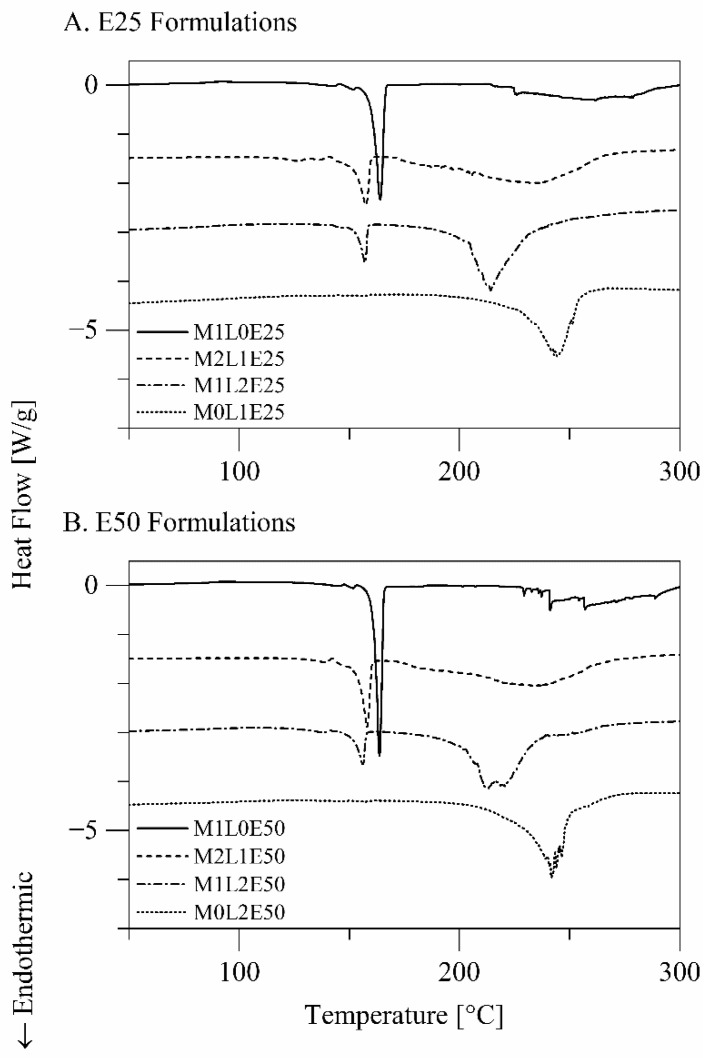
Differential scanning calorimetry thermograms of the powder formulations.

The differential scanning calorimetry thermograms of the formulations are presented in Figure 2. The formulations M1L0E25 and M1L0E50, which are composed of terbinafine hydrochloride and only mannitol as an excipient, exhibited a minor endothermic–exothermic transition at 140–154 °C and a sharp endothermic transition with *T*_peak_ = 163.83 ± 0.40 °C. The transitions, respectively, suggest that mannitol was present in the δ- and the α/β-crystalline forms in these formulations [19]. The formulations M0L1E25 and M0L1E50, which are composed of terbinafine hydrochloride and only leucine as an excipient, exhibited a broad endothermic transition with *T*_peak_ = 244.33 ± 2.43 °C, which corresponds to sublimation of l-leucine [20]. Combining mannitol and leucine as excipients in the formulations M2L1E25, M1L2E25, M2L1E50, and M1L2E50 shifted the endotherms corresponding to melting of δ- and α/β-mannitol and the endotherm corresponding to sublimation of l-leucine to lower temperatures. The mutual destabilization can be a result of mixing, where each excipient acted as an impurity in crystals of the other excipient. None of the formulations exhibited the endothermic peak characteristic of melting of crystalline terbinafine hydrochloride (*T*_peak_ = 200–205 °C [6]), suggesting that terbinafine hydrochloride was amorphous in all the formulations. The differential scanning calorimetry data do not suggest that the spray solvent composition influenced the crystallinities of the formulation ingredients.

### 3.2. Evaluation of Formulations

#### 3.2.1. Particle Disintegration and Dissolution

**Figure 3:**
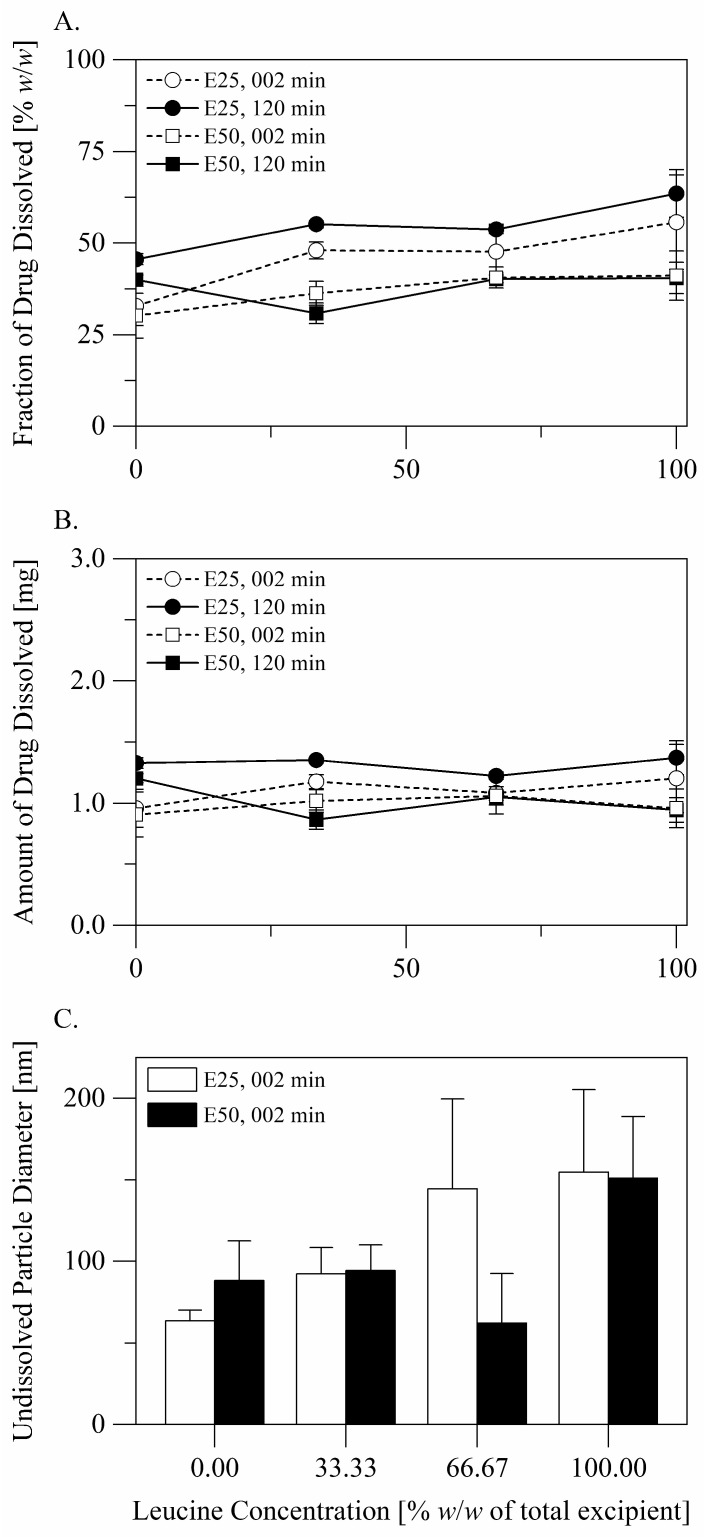
Disintegration and dissolution of the particles of the formulations: (**A**) the fraction of drug dissolved at 2 and at 120 min, (**B**) the amount of drug dissolved at 2 and at 120 min, (**C**) the diameter of undissolved drug particles at 2 min.

Disintegration and dissolution of the particles of the formulations are described in Figure 3. After dispersion of a dose into the disintegration medium, the excipient/s completely dissolve, whereas the drug partially dissolves. Plotting the fraction of the drug dissolved versus the concentration of leucine in the formulation (Figure 3A) implies that dissolution of the drug was influenced by the excipient composition. However, the differences between the fractions of the drug dissolved only reflected the differences between the drug concentrations in the formulations (see Table 2). Plotting the amount of the drug dissolved versus the concentration of leucine in the formulation (Figure 3B) makes it clear that neither the excipient composition nor the spray solvent composition influenced the amount of the drug dissolved after 2 min (two-way analysis of variance: *p* > 0.05). Changes in the amount of the drug dissolved with time were minor. It is plausible to conclude that dissolution of the drug was governed by saturation of the dispersion medium with the drug and that saturation was reached shortly after dispersion. For all the formulations, the average diameters of undissolved drug particles (Figure 3C) were smaller than 200 nm over the duration of the experiments.

#### 3.2.2. In Vitro Aerodynamic Performance (Cascade Impaction)

**Figure 4:**
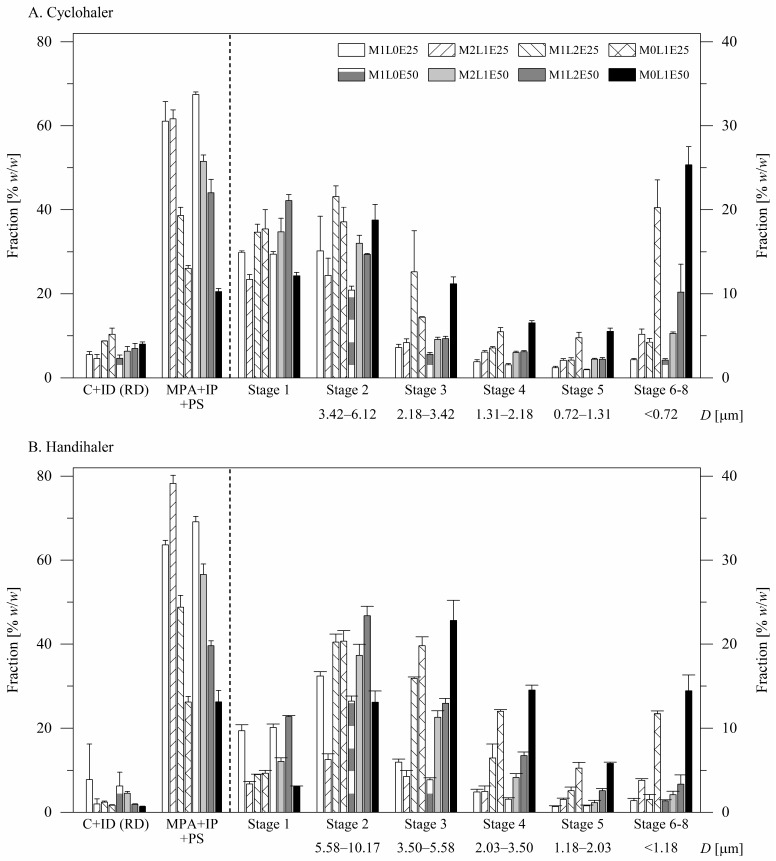
In vitro evaluation of the formulations by cascade impaction using (**A**) the Cyclohaler^®^ or (**B**) the Handihaler^®^ as an inhalation device. C+ID refers to the drug collected from the capsule shells and the inhalation device and is presented as a fraction of the recovered dose (left axis). MPA + IP + PS refers to the drug collected from the induction port, the mouthpiece adapter, and the preseparator and is presented as a fraction of the emitted dose (left axis). The drug collected from each of the impactor stages is presented as a fraction of the emitted dose (right axis). The size range of particles collected on each of the impactor stages is given to allow consideration of the impact of using different air flow rates for evaluation of the formulations using different inhalation devices.

**Figure 5:**
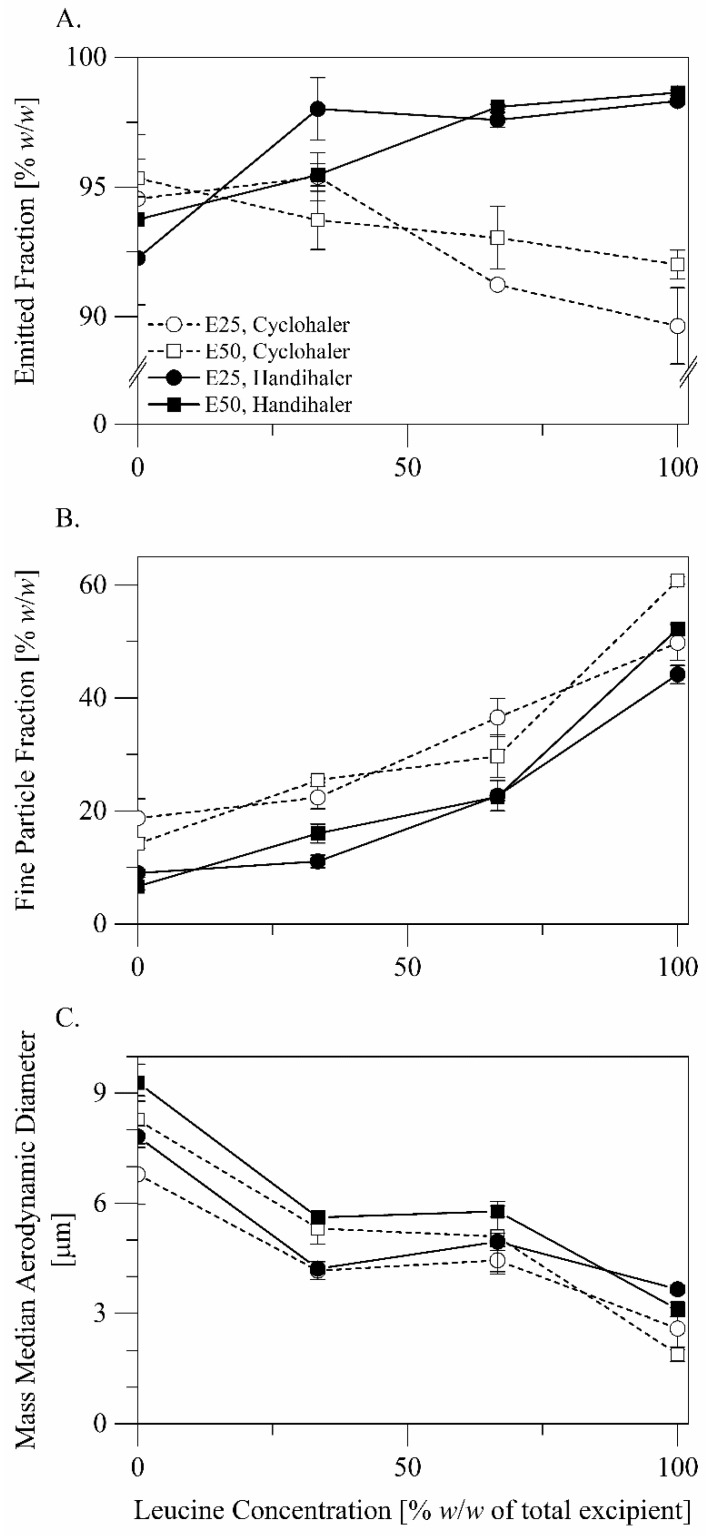
In vitro aerodynamic performance of the formulations: (**A**) the emitted fraction, (**B**) the fine particle fraction, (**C**) the mass median aerodynamic diameter.

The in vitro aerodynamic performance of the formulations is presented in Figure 4 and Figure 5. Results of statistical comparisons of the in vitro performance data are summarized in Table 3. The formulations exhibited mean emitted drug fractions (Figure 5A) greater than 89% *w*/*w*, suggesting good flow properties. The fine particle fractions (Figure 5B) of the formulations ranged from 6.64 ± 0.55% *w*/*w* for the formulation M1L0E50 when aerosolized by the Handihaler^®^ to 60.84 ± 0.67% *w*/*w* for the formulation M0L1E50 when aerosolized by the Cyclohaler^®^. The mass median aerodynamic diameters (Figure 5C) of the particles ranged from 9.29 ± 0.50 μm for the formulation M1L0E50 when aerosolized by the Handihaler^®^ to 1.90 ± 0.20 μm for the formulation M0L1E50 when aerosolized by the Cyclohaler^®^.

The excipient composition did not significantly influence particle emission from the inhalation devices (Figure 5A, three-way analysis of variance: *p* > 0.05) but influenced particle dispersibility, i.e., the fine fraction and the mass median aerodynamic diameter of the particles (Figure 5B,C, three-way analysis of variance: *p* < 0.001). As the concentration of leucine in the formulation increased, the fine particle fraction of the formulation increased (Tukey’s post hoc test: *p* < 0.001, pairwise). Replacing mannitol with leucine as a sole excipient was associated with a mean absolute increase in the fine particle fraction of 39.61% *w*/*w* (Tukey’s post hoc test: 95% CI = 37.74–41.48% *w*/*w*). Although using leucine was associated with reduced drug loading (Table 2), leucine improved particle dispersibility to a greater extent, leading to fine particle dose gain (Tukey’s post hoc test: *p* < 0.001, pairwise). Replacing mannitol with leucine as a sole excipient was associated with an increase in the mean normalized fine particle dose from 338 to 1102 μg (Tukey’s post hoc test: 95% CI = 722–806 μg). In agreement, as the concentration of leucine in the formulation increased, the mass median aerodynamic diameter of the formulation particles decreased (Tukey’s post hoc test: *p* < 0.001) except for the increase of the concentration of leucine from 33.33 to 66.67% *w*/*w* of the total excipient (Tukey’s post hoc test: *p* > 0.05). Replacing mannitol with leucine as a sole excipient was associated with a mean decrease in the mass median aerodynamic diameter of 5.23 μm (Tukey’s post hoc test: 95% CI = 4.82–5.63 μm). We can conclude that leucine was superior here as an excipient to mannitol and to mixtures of leucine and mannitol.

The spray solvent composition also did not significantly influence particle emission from the inhalation devices (Figure 5A, three-way analysis of variance: *p* > 0.05) and influenced particle dispersibility (Figure 5B,C, three-way analysis of variance: *p* < 0.01). The influences of the spray solvent composition on the fine particle fraction and the mass median aerodynamic particle diameter of the formulation varied in direction and magnitude with the concentration of leucine in the formulation. It is, however, noteworthy that the formulations involving only leucine as an excipient benefited in terms of the fine particle fraction from using the spray solvent with the higher ethanol concentration, i.e., 50% versus 25% *w*/*w* (Tukey’s post hoc test: mean absolute difference = 9.57% *w*/*w*, 95% CI = 6.40–12.73% *w*/*w*).

In terms of particle emission, the Handihaler^®^ and the Cyclohaler^®^ were similar for the formulations with a leucine concentration of 0.00–33.33% *w*/*w* of the total excipient (Tukey’s post hoc test: *p* > 0.05), but the Handihaler^®^ was generally superior to the Cyclohaler^®^ for the formulations with a leucine concentration of 66.67–100.00% *w*/*w* of the total excipient (Figure 5A, Tukey’s post hoc test: *p* < 0.05). In terms of particle dispersion, the Cyclohaler^®^ was superior to the Handihaler^®^ (Figure 5B, three-way analysis of variance: *p* < 0.001). This was true for all the formulations with a mean absolute improvement in the fine particle fraction of 9.17% *w*/*w* (Tukey’s post hoc test: 95% CI = 8.17–10.16% *w*/*w*). The fine particle fraction, FPF, is reported here as a fraction of the emitted dose. To consider the influences of the inhaler device on both particle emission and dispersion, we also compared the fine particle fractions of the recovered doses, FPF_RD_, calculated using the expression: FPF_RD_ = FPF × EF ⁄ 100. In terms of the fine particle fraction of the recovered dose, the Cyclohaler^®^ remained generally superior to the Handihaler^®^ (Tukey’s post hoc test: *p* < 0.001, 95% CI = 6.38–8.18% *w*/*w*). The formulations M0L1E25 and M0L1E50, which are composed of terbinafine hydrochloride and only leucine as an excipient, were exceptions. For these two formulations, the Handihaler^®^ advantage in emission was almost equivalent to the Cyclohaler^®^ advantage in dispersion, and the two inhaler devices were not significantly different in terms of the fine particle fraction of the recovered dose (Tukey’s post hoc test: *p* > 0.05). In terms of the mass median aerodynamic diameter, the Cyclohaler^®^ was generally similar to the Handihaler^®^. The Cyclohaler^®^ was moderately superior to the Handihaler^®^ only for the formulation M0L1E50 (Figure 5C, Tukey’s post hoc test: 95% CI = 0.09–2.33 μm). It is plausible to conclude that the Cyclohaler^®^ generally outperformed the Handihaler^®^ as an inhalation device for the developed formulations.

## 4. Discussion

A powder inhalation formulation of terbinafine, a broad-spectrum antifungal agent, has therapeutic potential in the management of pulmonary aspergillosis. To this end, we have earlier studied development of a powder inhalation formulation of terbinafine by spray drying [6]. The current study attempted to gain further insights into the spray drying process and to further optimize the developed formulations. More precisely, we aimed to (*i*) study the potential of l-leucine to improve the dispersibility of the formulation particles and (*ii*) compare the potentials of two inhalation devices to disperse the formulation particles.

Leucine is an amino acid that has desirable properties as an ingredient in spray-dried particles for dry powder inhalation [7]. Due to its surface-active properties, leucine has the potential to reduce the size of spray droplets. Additionally, due to its surface-active properties, leucine concentrates on the surfaces of spray droplets during drying. If leucine in a drying droplet starts to crystallize before other ingredients, it crystallizes in the form of a shell around the spray droplet. With further evaporation of the spray solvent, the shell deforms, folds, and/or collapses, resulting in a rugose particle with a corrugated surface [11,12,13,21]. The surface corrugations reduce the effective contact area of the particle and the magnitude of inter-particulate interactions [22]. Enrichment of the particle surface with leucine moreover reduces the surface energy of the particle [22]. Leucine is thereby used to enhance the dispersibility of spray-dried drug-containing particles for dry powder inhalation [4,7,8,9,10,11,12,13,14,23]. Leucine alone and in combination with other excipient materials is similarly used to develop dry powder inhalation carriers by spray drying [24]. Leucine furthermore provides protection for particles against humidity [9,11,14,25].

Using leucine in the current study as an excipient resulted in wrinkled particles with irregularly dimpled shapes (Figure 1, Section 3.1.2), consistent with the known spray drying behavior of leucine, suggesting that leucine contributed to the formation of crystalline (Figure 2, Section 3.1.3) particle shells. Using leucine as an excipient consequently resulted in particles with smaller aerodynamic diameters (Figure 5, Section 3.2.2). As the concentration of leucine in the formulation increased, the fine particle fraction and the fine particle dose of the formulation considerably increased, despite that the drug load decreased. The decreased drug load may be a consequence of particle fragmentation and surface perforation.

Leucine did not affect dissolution of terbinafine hydrochloride from the particles. The drug readily dissolved from all the formulations until saturation was reached (Figure 3, Section 3.2.1). Undissolved drug particles were smaller than 200 nm in diameter and thus have the potential to avoid clearance by alveolar macrophages and mucociliary escalation [26] and the potential to provide prolonged local action.

In the spray drying process, the spray solvent composition influences the interplay between spray generation, molecular diffusion, solvent evaporation, and solute precipitation/crystallization. The size of spray droplets generated by a vibrating-mesh atomizer, the atomizer generating spray in the Büchi B-90 nano spray dryer, can decrease as the viscosity of the spray solution increases [27]. The structure of spray-dried particles is controlled by the balance between solvent evaporation and solute diffusion kinetics. This can be illustrated by the Péclet number, Pe, concept [28]. The Péclet number is defined by the expression $Pe_{i}=\kappa /8D_{i}$, where $\kappa =d_{0}{}^{2}/\tau_{D}$ is the solvent evaporation rate, $D_{i}$ the solute *i* diffusion coefficient, $d_{0}$ the droplet diameter, and $\tau_{D}$ the droplet drying time. Increasing the Péclet number corresponds to increasing the rate of radial shrinkage of spray droplets relative to the rate of inward diffusion of solute molecules and results in particles with a relatively hollower structure and lower density. The simple Péclet number concept does not, however, account for surface activity, which drives molecules such as leucine toward rather than away from surfaces of spray droplets during drying. The concept also does not account for Péclet number changes that can take place during evaporation due to solute precipitation or due to changes in the composition of non-azeotropic cosolvent systems, such as the hydroethanolic co-solvent systems used here. The time, $\tau_{\mathrm{sat}}$, required for a solute *i* to reach saturation at the surface of a drying droplet is also controlled by the spray solvent composition [21] or more precisely by the solubility of the solute in the spray solvent (Equation (5)).

(5) $$\tau_{\mathrm{sat},i}=\frac{d_{0}{}^{2}}{\kappa }\left[1-{\left(E_{i}\frac{C_{0,i}}{C_{\mathrm{sat},i}}\right)}^{\frac{2}{3}}\right].$$

$C_{0}$ is the initial concentration of the solute and $C_{\mathrm{sat}}$ the solubility of the solute in the spray solvent. $E_{i}=C_{S,i}/C_{\mathrm{av},i}$ is the surface enrichment, defined as the ratio between the concentration of the solute at the surface and its average concentration in the drying droplet. Decreasing the solubility of the solute in the spray solvent promotes earlier precipitation of the solute and results in relatively larger and less dense particles [29]. When more than one solute is co-spray dried, promoting precipitation of one solute results in enrichment of surfaces of resulting particles with this solute.

In our earlier study, using a hydroethanolic (50.5% *w*/*w* ethanol) rather than an aqueous spray solvent for preparation of inhalable microparticles composed of terbinafine hydrochloride and mannitol by spray drying resulted in considerably smaller particles [6]. The Péclet number concept and the influence of the spray solvent on the solubility of mannitol, the major (90% *w*/*w*) solute, in the spray droplets do not explain this result. An influence of the spray solvent on the size of spray droplets provided a possible explanation. The hydroethanolic solvent was more viscous than the aqueous solvent and may have thus resulted in smaller spray droplets. The influence of the spray solvent on the solubility of terbinafine hydrochloride in the spray droplets provides another possible explanation. In contrast to mannitol, terbinafine hydrochloride was more soluble in the hydroethanolic spray solvent than in the aqueous spray solvent [30]. Using a hydroethanolic rather than an aqueous spray solvent delays precipitation of terbinafine hydrochloride in spray droplets during drying. At least when an aqueous spray solvent was used, the size of the particles was apparently determined by the onset of terbinafine hydrochloride precipitation during drying. Enrichment of the particle surfaces with terbinafine hydrochloride when an aqueous spray solvent was used could not be detected by energy-dispersive X-ray spectroscopy, but it apparently impaired the dispersibility of the particles.

In the current study, we investigated the influences of the concentration of ethanol (50% versus 25% *w*/*w*) in the spray solvent on the characteristics of the spray-dried particles. The influences of the concentration of ethanol in the spray solvent on the fine fraction and the mass median aerodynamic diameter of the particles varied in direction and magnitude with the concentration of leucine in the particles but were generally minor. Remarkably, however, increasing the concentration of ethanol in the hydroethanolic spray solvent improved the fine particle fraction of the formulations involving only leucine as an excipient (Figure 5B). This was not associated with a significant influence on the mass median aerodynamic diameter of the particles. Leucine is less soluble in ethanol than in water [8]. Increasing the concentration of ethanol in the spray solvent improved the dispersibility of the particles potentially by promoting enrichment of the particle surfaces with leucine. Remarkably, increasing the concentration of ethanol in the hydroethanolic spray solvent increased the drug loading efficiency. This can also be attributed to enrichment of the particle surfaces with leucine and to the delayed onset of terbinafine hydrochloride precipitation during drying.

The Handihaler^®^ is characterized by a higher resistance than the Cyclohaler^®^. The pressure drop generated across the device during aerosolization is thus higher for the Handihaler^®^ than for the Cyclohaler^®^. On the other hand, the capsule aperture size is smaller in the Cyclohaler^®^ than in the Handihaler^®^ and can thus generate stronger shear forces during capsule emptying [31]. In the current study, the Handihaler^®^ and the Cyclohaler^®^ exhibited generally similar potential to emit spray-dried microparticles. The Handihaler^®^, however, emitted the formulations with a leucine concentration of 66.67–100.00% *w*/*w* of the total excipient more efficiently than the Cyclohaler^®^. On the other hand, the Cyclohaler^®^ exhibited greater potential to disperse the spray-dried microparticles than the Handihaler^®^. The difference in particle emission is potentially linked to the difference in the pressure drop generated across the device during aerosolization, whereas the difference in particle dispersion is linked to the difference in shear forces generated during capsule emptying.

## 5. Conclusions

Leucine had the potential to improve the performance of spray-dried terbinafine microparticles for inhalation. When leucine was used as an excipient, using 50% *w*/*w* ethanol in water as a spray solvent further improved the dispersibility of the spray-dried microparticles, potentially via promoting enrichment of the particle surfaces with leucine. The Cyclohaler^®^ outperformed the Handihaler^®^ as an inhalation device for the spray-dried microparticles.

## Figures and Tables

**Table 1:** The design of the spray drying experiments.

Formulation *^a^*	Spray Solution Composition *^b^* [% *w*/*w* in Deionized Water]				Nominal Particle Composition [% *w*/*w*]		
	Drug	Mannitol	Leucine	Ethanol	Drug	Mannitol	Leucine
M1L0E25	0.113	0.638	0.000	24.813	15.00	85.00	0.00
M2L1E25	0.113	0.425	0.212	24.813	15.00	56.70	28.31
M1L2E25	0.113	0.212	0.425	24.813	15.00	28.31	56.70
M0L1E25	0.113	0.000	0.638	24.813	15.00	0.00	85.00
M1L0E50	0.113	0.638	0.000	49.625	15.00	85.00	0.00
M2L1E50	0.113	0.425	0.212	49.625	15.00	56.70	28.31
M1L2E50	0.113	0.212	0.425	49.625	15.00	28.31	56.70
M0L1E50	0.113	0.000	0.638	49.625	15.00	0.00	85.00

*^a^* Formulation codes are expressed in the form M*x*L*y*E*z*, where *x*:*y w*/*w* is the mannitol:leucine ratio and *z*% *w*/*w* is the concentration of ethanol in the spray solvent. *^b^* All spray solutions contained hydrochloric acid at a concentration of 0.01 mol/kg. For all formulations, the total solute concentration in the spray solution was 0.75% *w*/*w* and the nominal drug:total excipient ratio was 15:85 *w*/*w*.

**Table 2:** The yields of the spray drying experiments.

Formulation	Yield *^a^* [% *w*/*w*]	Loading Efficiency *^b^* [% *w*/*w*]	Drug Recovery [% *w*/*w*]
M1L0E25	44.6 ± 1.4	97.2 ± 3.0	43.3 ± 1.9
M2L1E25	51.4 ± 13.2	81.7 ± 5.3	42.0 ± 11.1
M1L2E25	67.7 ± 10.7	75.9 ± 2.3	51.4 ± 8. 3
M0L1E25	59.8 ± 7.2	71.9 ± 3.8	43.0 ± 5.6
M1L0E50	47.7 ± 11.1	100.2 ± 4.7	47.8 ± 11.4
M2L1E50	56.0 ± 10.0	93.5 ± 1.3	52.3 ± 9.4
M1L2E50	60.4 ± 0.3	87.0 ± 1.2	52.6 ± 0.8
M0L1E50	60.8 ± 5.7	77.8 ± 2.2	47.3 ± 4.6

*^a^* Averages ± standard deviations (*N* = 2). *^b^* Averages ± standard deviations (*N* = 4). The nominal drug load was 15% *w*/*w* for all formulations.

**Table 3:** Analysis of the influences of the excipient composition, the spray solvent composition, and the inhalation device on the in vitro aerodynamic performance of the spray-dried formulation *^a^*.

Factor	Emitted Fraction	Fine Particle Fraction	MMAD *^b^*
Excipient (leucine concentration)	NS	S ***	S ***
Spray solvent (ethanol concentration)	NS	S **	S ***
Inhaler (Handihaler vs. Cyclohaler)	S ***	S ***	S ***
Excipient × Solvent	NS	S ***	S ***
Excipient × Inhaler	S ***	NS	NS
Spray solvent × Inhaler	NS	NS	NS

*^a^* The influences were analyzed by three-way analysis of variance. The significance of each influence is indicated by one of the following abbreviations: NS: not significant, S **: significant at *p* < 0.01, S ***: significant at *p* < 0.001. *^b^* Mass median aerodynamic diameter.

## Data Availability

Not applicable.
